# Important Requirements for Desorption/Ionization Mass Spectrometric Measurements of Temozolomide-Induced 2′-Deoxyguanosine Methylations in DNA

**DOI:** 10.3390/cancers15030716

**Published:** 2023-01-24

**Authors:** Margaux Fresnais, Ina Jung, Uli B. Klein, Aubry K. Miller, Sevin Turcan, Walter E. Haefeli, Jürgen Burhenne, Rémi Longuespée

**Affiliations:** 1 Department of Clinical Pharmacology and Pharmacoepidemiology, Heidelberg University Hospital, Im Neuenheimer Feld 410, 69120 Heidelberg, Germany; 2 Cancer Drug Development, German Cancer Research Center (DKFZ), Im Neuenheimer Feld 280, 69120 Heidelberg, Germany; 3 Neurology Clinic and National Center for Tumor Diseases, Heidelberg University Hospital, Im Neuenheimer Feld 460, 69120 Heidelberg, Germany

**Keywords:** temozolomide, 2′-deoxyguanosine, DNA methylation, mass spectrometry, desorption/ionization

## Abstract

**Simple Summary:**

Monitoring chemical action of drugs directly at their molecular target would be particularly valuable for personalized medicine. In temozolomide-exposed biological samples, 2′-deoxyguanosines and O6-methylated species are compounds of interest to monitor chemical effects. However, their analysis can be hampered by molecular interferences. Hereby, desorption/ionization mass spectrometry was evaluated for such investigation. Here, we illustrate that, without following specific requirements in terms of sample preparation and mass spectrometric instrumentation, these analyses are prone to important artefacts.

**Abstract:**

In clinical pharmacology, drug quantification is mainly performed from the circulation for pharmacokinetic purposes. Finely monitoring the chemical effect of drugs at their chemical sites of action for pharmacodynamics would have a major impact in several contexts of personalized medicine. Monitoring appropriate drug exposure is particularly challenging for alkylating drugs such as temozolomide (TMZ) because there is no flow equilibrium that would allow reliable conclusions to be drawn about the alkylation of the target site from plasma concentrations. During the treatment of glioblastoma, it appears, therefore, promising to directly monitor the alkylating effect of TMZ rather than plasma exposure, ideally at the site of action. Mass spectrometry (MS) is a method of choice for the quantification of methylated guanines and, more specifically, of O6-methylguanines as a marker of TMZ exposure at the site of action. Depending on the chosen strategy to analyze modified purines and 2′-deoxynucleosides, the analysis of methylated guanines and 2′-deoxyguanosines is prone to important artefacts due to the overlap between masses of (i) guanines from DNA and RNA, and (ii) different methylated species of guanines. Therefore, the specific analysis of O6-methyl-2′deoxyguanosine, which is the product of the TMZ effect, is highly challenging. In this work, we report observations from matrix-assisted laser desorption/ionization (MALDI), and desorption electrospray ionization (DESI) MS analyses. These allow for the construction of a decision tree to initiate studies using desorption/ionization MS for the analysis of 2′-deoxyguanosine methylations induced by TMZ.

## 1. Introduction

In clinical pharmacology, approaching the anatomical, cellular, and molecular sites of action (SOAs) of drugs allows to monitor the ultimate chemical and biological actions of a therapeutic compound. However, measuring the concentration of drugs covalently bound to their molecular SOA can be highly challenging [1]. In addition, in some contexts, the biological action is not driven by the covalent interaction of molecular SOA with the drug itself, but with a metabolite that is too instable to be quantified directly, as for temozolomide (TMZ). TMZ is the standard therapy for the treatment of glioblastoma in conjunction with tumor-removing surgery [2,3]. At the physiological pH, TMZ decays into the methyldiazonium ion [4,5], which methylates nucleophilic sites of DNA [5] and RNA [6,7], including the O6 position of guanines [5]. Although representing only 5% of all DNA methylations, O6-methylguanine (O6-mG) is responsible for the biological action of TMZ in methylguanine methyltransferase (MGMT)-inactive tumor cells [8,9,10], e.g., in the presence of isocitrate dehydrogenase (IDH) mutations [11,12,13,14]. The major methylated species of guanines (i.e., N7-mG) is repaired by the base excision repair system and then rarely leads to a pharmacological response to TMZ [10]. Measuring pharmacologically relevant modifications of DNA induced by TMZ would, therefore, consist of the specific quantification of O6-mG. As recently illustrated, depending on the chosen combination of sampling strategy, sample preparation, and analytical method, this task can be challenged by the presence of RNA in biological samples and by N7-mG species [8]. Since N7-mG and O6-mG species bear the same mass, separative methods are necessary to distinguish N7-mG and O6-mG species, using liquid chromatography [15,16] and/or ion mobility before and after ionization, respectively [8]. O6-mG species from DNA and RNA are exactly the same compounds. Therefore, it is impossible to specifically quantify O6-mG from DNA in biological samples containing both DNA and RNA without RNA removal [8].

On-surface mass spectrometric (MS) analyses of solid samples (e.g., tissue section) in imaging mode [17,18] would offer the unique advantage to allow correlation between guanine modifications and histological information. However, on-surface analyses require sample preparation methods that preserve the histological context [19]. Therefore, tissue homogenization is not possible and washing steps in order to remove RNA should be avoided. Additionally, using desorption/ionization (DI)-MS, separation of O6-mG from N7-mG is not possible before their ionization together with other compounds of the biological matrix. For these reasons, the analyses of 2′-deoxyguanosines (2dGO) and their active methylated counterpart O6-methyl-2′deoxyguanosine (O6-m2dGO) would allow for more flexibility in terms of sample preparation for DNA digestion because any digestion mix would result in digestion products differing between DNA and RNA [8]. In addition, the possibility to use ion mobility (IM) with DI-MS instrumentations would represent a valuable option to separate O6-m2dGO from N7-m2dGO [20]. In the present white paper, we present MS analyses of pure solutions of guanine, 2dGO, and O6-m2GO using DI sources, namely matrix-assisted laser desorption/ionization (MALDI) and desorption electrospray ionization (DESI). First, we show that different MALDI matrices display different ionization capacities for both guanines and 2dGO. Second, and more importantly, we demonstrate that MALDI and DESI analyses can lead to dominant in-source and post-source decay (ISD and PSD, respectively) events requiring careful choices of sample preparation and analytical procedures to establish successful targeted quantification assays. Based on these observations, we provide appropriate recommendations for the future establishment of DI-MS-based workflows for the analysis of 2dGO and O6-m2dGO.

## 2. Material and Methods

### 2.1. Chemicals

MS-grade water, organic solvents, and formic acid (FA) were purchased from Biosolve Chimie SARL (Dieuze, France). Alpha-cyano-4-hydroxy-cinnamic acid (CHCA), 2,5-dihydroxybenzoic acid (2,5-DHB), trifluoroacetic acid (TFA), 3-hydroxypicolinic acid (3-HPA), graphene, red phosphorus, and guanine were purchased from Sigma-Aldrich (Darmstadt, Germany). 2dGO, O6-m2dGO and N7-mG were purchased from Toronto Research Chemicals (North York, ON, Canada). 2,3,4,5-tetrakis(3′,4′-dihydroxylphenyl)thiophene (DHPT) was synthesized as described in the following paragraph.

### 2.2. Synthesis of DHPT

The synthesis is a slightly modified version of a previously published procedure [21] that could not directly be reproduced.

In a 100 mL Schlenk flask, tetrabromothiophene (1.2053 g, 3.015 mmol, 1.00 eq) and Pd(PPh_3_)_4_ (218.3 mg, 0.189 mmol, 6 mol%) were dissolved in degassed (15 min N_2_ sparge) dioxane (12 mL) under an atmosphere of argon. After 15 min, 3,4-dimethoxyphenylboronic acid (2.7342 g, 15.025 mmol, 4.98 eq) was added to the yellow solution. An additional 3 mL of degassed dioxane was used to rinse all material down the walls of the flask. To the resulting suspension was added an aqueous solution of K_2_CO_3_ (2.0 M, 1.5 mL, 3.0 mmol, 1.0 eq), which itself had been sparged with N_2_ gas for 15 min. This mixture was submerged into a pre-heated 80 °C oil bath. After 3 h, starting material was still apparent by TLC (30% EtOAc in hexanes), so additional base (4M K_2_CO_3_, 3.0 mL, 12.0 mmol, 4 eq) was added. The resulting biphasic mixture was stirred vigorously at 80 °C. After 22 h, the starting material was consumed and the reaction was cooled to room temperature, diluted with H_2_O (150 mL) and EtOAc (150 mL), and the two layers were separated. The aqueous layer was extracted with EtOAc (2 × 50 mL), the combined organic layers were washed with brine (25 mL), dried (MgSO_4_), filtered, concentrated, and dried in vacuo. The product was purified by automated chromatography (20% to 50% EtOAc gradient in hexanes) to give a diarylated product, which, based on symmetry considerations from the NMR spectra, is either 2,5-dibromo-3,4-bis(3,4-dimethoxyphenyl)thiophene or 3,4-dibromo-2,5-bis(3,4-dimethoxyphenyl)thiophene. The product was obtained as a white solid (1.258 g, 81% yield).

The NMR properties of the product compound were the following: ^1^H NMR (CDCl_3_, 400 MHz) δ 7.23–7.19 (m, 4H), 6.96–6.92 (m, 2H), 3.94 (s, 6H), and 3.93 (s, 6H) ppm.

In a 100 mL Schlenk flask, the diarylated product from the previous step (1.255 g, 2.44 mmol, 1.0 eq), 3,4-dimethoxyphenylboronic acid (1.333 g, 7.32 mmol, 3.00 eq), and Pd(PPh_3_)_4_ (734 mg, 0.635 mmol, 26 mol%) were dissolved in degassed dioxane (30 mL) under argon. Then, aqueous K_2_CO_3_ (4 M, 2.4 mL, 9.6 mmol, 3.9 eq) was added. The resulting mixture was submerged in a preheated 80 °C oil bath. After 18 h, additional catalyst (60 mg) and boronic acid (300 mg) were added and the temperature of the oil bath was increased to 100 °C. After another 72 h, the starting material was consumed and largely one product had formed. The reaction was cooled to room temperature and diluted with H_2_O (200 mL) and EtOAc (150 mL). The two layers were separated and the aqueous layer was extracted with EtOAc (2 × 75 mL). The combined organic layers were washed with brine (30 mL), dried (MgSO_4_), filtered, and concentrated in vacuo. The product was purified by automated column chromatography (40% to 100% EtOAc gradient in hexanes), followed by recrystallization from an EtOAc/hexane mixture to give 2,3,4,5-tetrakis(3,4-dimethoxyphenyl)thiophene (1.124 g, 73%) as a gray powder.

The NMR properties of the product compound were the following: ^1^H NMR (CDCl_3_, 400 MHz) δ 6.92 (dd, *J* = 8.3, 2.1 Hz, 1H), 6.77 (d, *J* = 8.3 Hz, 1H), 6.73 (d, J = 2.1 Hz, 1H), 6.68 (d, *J* = 8.2 Hz, 1H), 6.58 (dd, *J* = 8.2, 1.9 Hz, 1H), 6.52 (d, *J* = 1.9 Hz, 1H), 3.86 (s, 3H), 3.82 (s, 3H), and 3.59 (s, 3H), 3.52 (s, 3H) ppm.

To a solution of 2,3,4,5-tetrakis(3,4-dimethoxyphenyl)thiophene (1.115 g, 1.773 mmol, 1.0 eq) in anhydrous CH_2_Cl_2_ (30 mL), was added a solution of BBr_3_ (32 mL, 1.0 M in CH_2_Cl_2_, 18 eq) at –78 °C under argon. The resulting wine-colored solution was stirred at this temperature for 1 h and at 0 °C for 3 h. After cooling to –78 °C, the reaction mixture was quenched with MeOH (10 mL) and concentrated to an oil. The mixture was suspended in H_2_O (30 mL) and heated in a 70 °C oil bath for 1 h, during which time a white precipitate formed. The mixture was cooled to 0 °C, filtered, and the solid was washed with H_2_O (4 × 30 mL), and air dried. The resulting powder was dissolved in acetone, concentrated, and then dried on a lyophilizer to give DHPT as a gray powder (650 mg, 71%). Spectroscopic data were consistent with the literature [22].

### 2.3. Sample Preparation

Stock solutions of guanine, 2dGO, O6-m2dGO, and N7-mG were prepared at respective concentrations: 1.32 mg/mL, 1.56 mg/mL, 0.302 mg/mL, and 1.5 mg/mL, all dissolved in solutions of MeOH:H_2_O 1:1. For MALDI analyses, 0.5 µg/mL solutions were created from the stock solutions and 1 µL was deposited on a MALDI metal target [23], followed by the deposition of 1 µL of the MALDI matrix solution at the following concentration and solvent mixtures: graphene 2 mg/mL in EtOH/TFA 0.1%, DHPT 10 mg/mL in MeOH/TFA 0.1%, 3-HPA 50 mg/mL in H_2_O, DHB 90 mg/mL in MeOH/H_2_O/TFA 1:1:0.1%, CHCA 25mg/mL MeOH/H_2_O/TFA 1:1:0.1%. For DESI analyses, 1 µL of the 0.5 mg/mL solutions were deposited on spots of Aquarray DMA Slides G-dd-202 (Aquarray GmbH, Eggenstein-Leopoldshafen, Germany) for further direct analyses, as described before [24].

### 2.4. Mass Spectrometric Analyses

**Figure 1:**
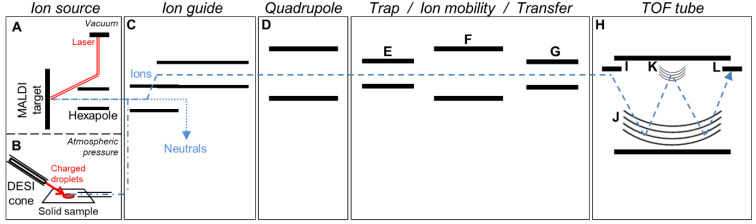
Schematic representation of the Waters Synapt G2-Si device. Matrix-assisted laser desorption/ionization (MALDI) source with laser, MALDI target and hexapole ion guide (**A**) and desorption electrospray ionization (DESI) source with DESI sprayer, solid sample, and transfer line (**B**). Off-axis ion guide (**C**), quadrupole for ion selection (**D**). Trap cell for collision-induced dissociation (CID) or ion selection (**E**), travelling wave ion mobility cell (**F**), transfer cell for CID or ion selection (**G**). Time of flight (TOF) tube (**H**) consisting of a high field pusher (**I**), a dual stage reflectron (**J**), an ion mirror for resolution (W) mode (**K**), and an ion detection system (**L**). The laser beam and charged droplet spray responsible for the ionization processes are shown in red solid lines, and the paths of neutrals and ions are shown in blue dotted lines.

Analyses were performed on a Synapt G2-Si instrument (Figure 1, Waters Corp, Milford, MA, USA) consisting of orthogonal acceleration (oa)-quadrupole (Q)-ion mobility (IM)-time-of-flight (TOF) MS equipped with either a MALDI source (Figure 1A) or a DESI source (Figure 1B) and controlled under MassLynx v4.1 (Waters Corp), as fully described previously [20]. The instrument was used in positive and resolution mode (“W” mode, Figure 1H). We previously described the IM-MS parameters for the analysis of native compounds (no selection of the native compounds or selection of a specific native ion in Q (Figure 1D), followed by IM separation (Figure 1F) before MS detection (Figure 1H), referred as Methods 2 and 3, respectively in [20] and detailed in Table 1) and IM-MS/MS parameters for the analysis of fragments (selection of specific native ions in Q followed by collision-induced dissociation at 20–25 V (Figure 1E) and subsequent IM separation of the fragments before MS detection, referred as Method 4 in [20,23] and detailed in Table 1). Related acquisition methods applied in this study are further detailed in Table 1.

The MALDI-MS analyses were performed as described before [23] using a spiral pattern, and DESI-MS analyses were performed using parameters previously described for the high-performance DESI sprayer [24].

### 2.5. Data Processing

MS spectra and mobilograms were extracted from MassLynx v4.1. Recommendations for reporting results of IM-MS measurements [25] were followed. Because IM is used here as a separation method and not for structural analyses, the drift times (DT) are reported as IM spectrometry (IMS) data. The previously described MobA method [20] was used for data extraction; the mobility peaks specific to the compounds of interest (extracted ion mobilograms (XIM)) were extracted from their specific m/z peak in the combined mass spectra. The obtained XIM were then automatically integrated to retrieve the peak areas of the targeted compounds using MassLynx software [20,23].

## 3. Results

### 3.1. MALDI-MS Analyses of Guanine and 2′-Deoxyguanosine

**Figure 2:**
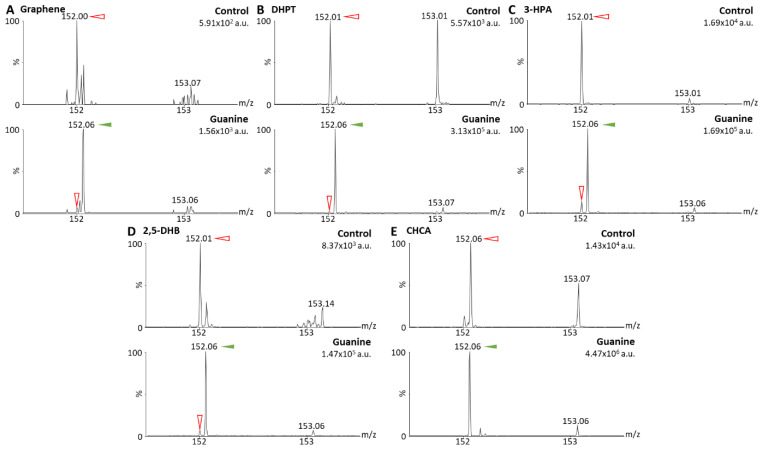
Matrix-assisted laser desorption/ionization (MALDI) mass spectra of solvent (control) and guanine solutions at 0.5 µg/mL in MeOH/H_2_O 1:1 obtained with graphene as MALDI matrix (**A**), 2,3,4,5-tetrakis(3′,4′-dihydroxylphenyl)thiophene (DHPT) (**B**), 3-hydroxypicolinic acid (3-HPA) (**C**), 2,5-dihydroxybenzoic acid (2,5-DHB) (**D**), and alpha-cyano-4-hydroxy-cinnamic acid (CHCA) (**E**). Spectra were acquired using method Q-152 (positive resolution mode, with quadrupole focused on m/z 152). Maximum intensities are given in arbitrary units (a.u.) on the top right corner of each spectrum. Peaks from the MALDI matrices are marked with red open arrows and peaks from guanine (m/z 152.06) with green solid arrows.

**Figure 3:**
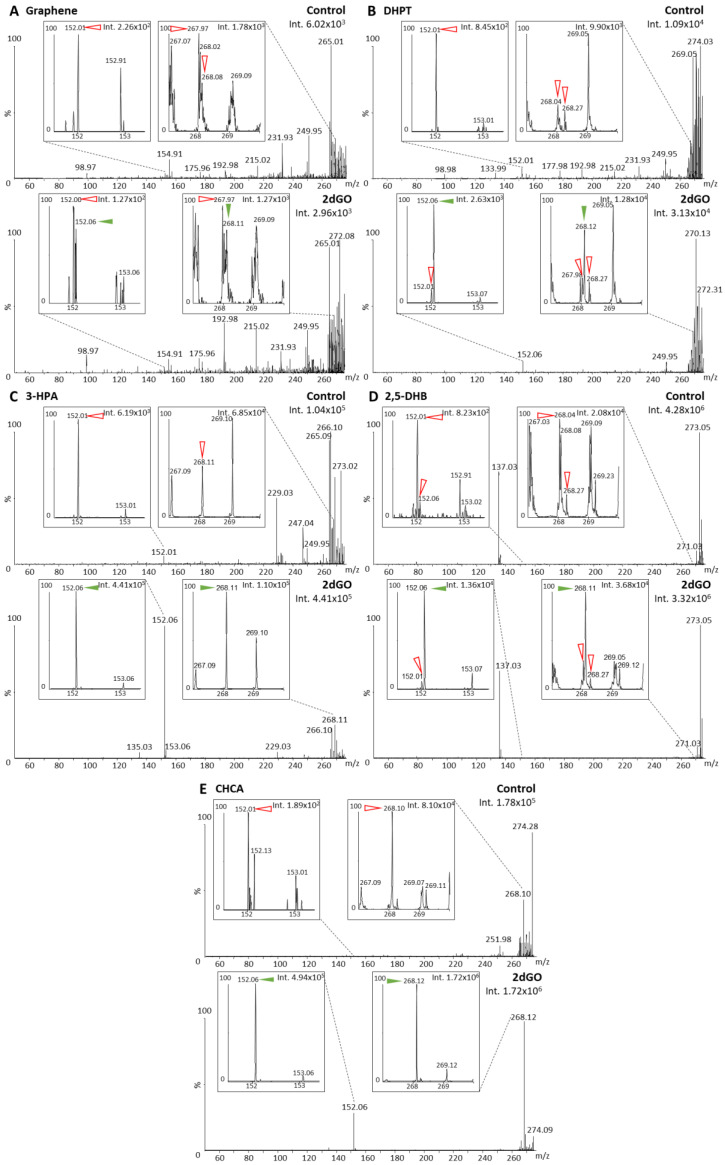
Matrix-assisted laser desorption/ionization (MALDI) mass spectra of solvent (control) and 2′-deoxyguanosine (2dGO) solution at 0.5 µg/mL in MeOH/H_2_O 1:1 with zoomed views on the 2dGO parent peak at m/z 268.11 and its fragment peak at m/z 152.06. Spectra were obtained with graphene MALDI matrix (**A**), 2,3,4,5-tetrakis(3′,4′-dihydroxylphenyl)thiophene (DHPT) (**B**), 3-hydroxypicolinic acid (3-HPA) (**C**), 2,5-dihydroxybenzoic acid (2,5-DHB) (**D**), and alpha-cyano-4-hydroxy-cinnamic acid (CHCA) (**E**). Spectra were acquired using method Q-268 (positive resolution mode, with quadrupole focused on m/z 268). Maximum intensities are given in arbitrary units (a.u.) on the top right corner of each spectrum and zoomed view. Peaks from the MALDI matrices are marked with red open arrows and peaks from 2dGO with green solid arrows.

For initial ionization tests with MALDI-MS, non-methylated guanine (Figure 2) and 2dGO (Figure 3) were tested using five different MALDI matrices: (i) Graphene, a MALDI matrix allowing for interference-free analysis of small molecules and demonstrated to be efficient for the analyses of guanines and 2dGO [26]; (ii) DHPT, developed for the analyses of low-mass amines including guanines [21]; (iii) 3-HPA, reported for the ionization of oligonucleotides [27]; (iv) 2,5-DHB [20] and (v) CHCA [28], two MALDI matrices universally used for small compounds. Initial analyses of non-methylated guanine and 2dGO were performed using methods Q-152 and Q-268 (Table 1).

Guanine (parent peak at m/z 152.06) gave a clear signal with four out of five MALDI matrices (i.e., intensities > 1.10^5^ arbitrary units (a.u.)) (Figure 2B–E), and only a weak signal could be detected with graphene (i.e., 1.56 × 10^3^ a.u., Figure 2A). For 2dGO, the parent peak at m/z 268.11 was only weakly detected with intensities close to those of interfering signals in the control analyses for graphene (9.62 × 10^2^ a.u. vs. 1.78 × 10^3^ a.u., Figure 3A), DHPT (9.55 × 10^3^ a.u. vs. about 3.0 × 10^3^ a.u., Figure 3B), and 2,5-DHB (3.68 × 10^4^ a.u. vs. 2.08 × 10^4^ a.u., Figure 3D). Using 3-HPA, the m/z 268.11 peak was detected for both the control sample and the 2dGO sample (Figure 3C), with a higher intensity in the 2dGO sample (i.e., 1.10 × 10^5^ a.u., twice the intensity of the 3-HPA peak at m/z 269.10) compared to the intensity in the control sample (i.e., 3.52 × 10^4^ a.u., half the intensity of 3-HPA peak at m/z 269.10). As for guanine samples, the highest signal for 2dGO was obtained with the CHCA MALDI matrix (1.72 × 10^6^ a.u. vs. 8.10 × 10^4^ a.u. in control, Figure 3E). The analysis of 2dGO also revealed the presence of another peak at m/z 152 corresponding to the guanine moiety of the compound, thus revealing fragmentation processes during the analyses. Because the quadrupole was set to the parent mass for the analysis (i.e., focus on m/z 268 for the analysis of 2dGO), it seems that the fragmentation event took place in or after the quadrupole (Figure 1D), and could, thus, be described as PSD. The fragment signal was visible for all MALDI matrices, even when the parent compound was detected with a weak signal (Figure 3) but PSD events had different impact depending on the MALDI matrix. Using graphene, the intensity of the fragment at m/z 152 was only 10% of the parent intensity (Figure 3A). In DHPT, 2,5-DHB, and CHCA, the fragment ion represented 28%, 37%, and 29% of the parent peak, respectively (Figure 3B,D,E). The PSD event was dominant with 3-HPA, with a 4-fold higher intensity for the fragment peak compared to the parent peak (Figure 3C).

**Figure 4:**
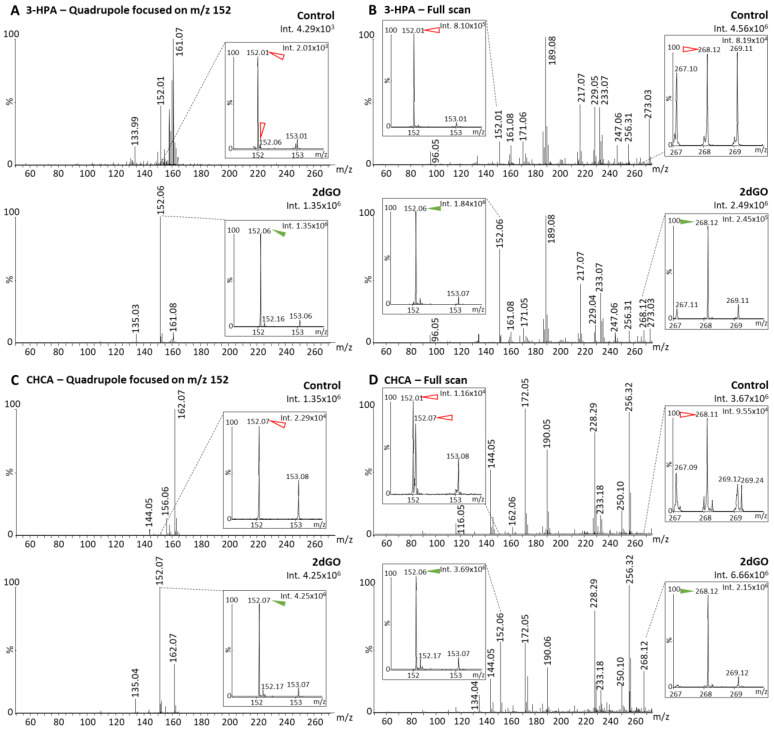
Matrix-assisted laser desorption/ionization (MALDI) mass spectra of solvent (control) and 2′-deoxyguanosine (2dGO) stock solution at 0.5 µg/mL in MeOH/H_2_O 1:1 with zoomed views on the 2dGO parent peak at m/z 268.1 and its fragment peak at m/z 152.06. Spectra were obtained with 3-hydroxypicolinic acid (3-HPA) MALDI matrix with the quadrupole focused on the fragment mass m/z 152 (**A**) or in full scan (**B**), and with alpha-cyano-4-hydroxy-cinnamic acid (CHCA) MALDI matrix with the quadrupole focused on the fragment mass m/z 152 (**C**) or in full scan (**D**). Maximum intensities are given in arbitrary units (a.u.) on the top right corner of each spectrum and zoomed view. Peaks from the MALDI matrices are marked with red open arrows and peaks from 2dGO with green solid arrows.

In order to investigate whether fragmentations could also happen before the quadrupole selection, further analyses were performed using the two best MALDI matrices (i.e., 3-HPA and CHCA) with the full scan and the Q-152 methods (Table 1, Figure 4). With method Q-152, 3.1-fold and 8.6-fold higher intensities could be detected for the fragment m/z 152 using 3-HPA (Figure 4A) and CHCA (Figure 4C), respectively, compared to method Q-268, thus suggesting that fragmentation events mainly occur before the quadrupole. This process could take place during the ionization (i.e., ISD, Figure 1A) or after, in the hexapole (Figure 1A) or the ion guide (Figure 1C). This was confirmed by analyses with the full-scan method showing predominant fragment peaks: (i) Compared to the fragment peaks obtained with method Q-268 (i.e., 4.2-fold and 7.5-fold higher intensities using 3-HPA (Figure 4B) and CHCA (Figure 4D), respectively); (ii) compared to the parent peak at m/z 268.12 on the same mass spectrum (i.e., 7.5-fold and 1.7-fold higher intensities for the fragment using 3-HPA (Figure 4B) and CHCA (Figure 4D), respectively). Overall, these results indicated that fragmentation events were dominant as compared to the stable compounds and were mainly taking place early in the ion path (i.e., mainly in the source, Figure 1A,C).

### 3.2. DESI Analyses of 2′-Deoxyguanosine, O6-Methyl-2′-Deoxyguanosine

ISD and PSD events are well known in MALDI-MS, where the fluence of the laser plays a major role [29,30]. In order to test whether these events could be avoided using another DI-MS method, 2dGO was also analyzed by DESI-MS, as well as O6-m2dGO, the critical compound to monitor TMZ action.

**Figure 5:**
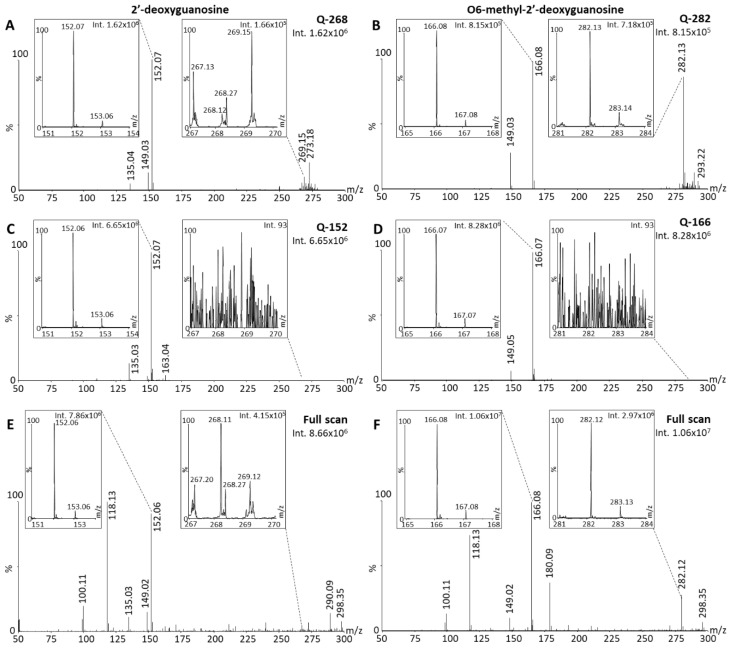
Desorption electrospray ionization (DESI) mass spectra of 2′-deoxyguanosine (2dGO) and O6-methyl-2′-deoxyguanosine (O6-m2dGO) stock solutions at 0.5 µg/mL in MeOH/H_2_O 1:1 with zoomed views on the parent peaks (m/z 268.11 and m/z 282.12, respectively) and the main post-source decay (PSD) fragment peaks (m/z 152.06 and m/z 166.08, respectively). Spectra were acquired with the quadrupole focused on the parent mass without collision energy; methods Q-268 for 2dGO (**A**) and Q-282 for O6-m2dGO (**B**), with the quadrupole focused on the PSD fragment mass without collision energy; methods Q-152 for 2dGO (**C**) and Q-166 for O6-m2dGO (**D**), or in full scan for both 2dGO (**E**) and O6-m2dGO (**F**). Maximum intensities are given in arbitrary units (a.u.) on the top right corner of each spectrum and zoomed view.

Using the parent-focused methods Q-268 and Q-282 (Table 1), DESI-MS data demonstrated that 2dGO (Figure 5A) and O6-m2dGO (Figure 5B) were also instable using DI under ambient conditions, with dominant ISD/PSD fragments compared to the native compounds (10.8-fold higher intensity of the m/z 152 peak compared to the m/z 268 peak for 2dGO, and 1.1-fold higher m/z 166 peaks compared to m/z 282 peaks for O6-m2dGO). The analysis of the compounds with methods Q-152 and Q-166 (Table 1) resulted in a 4.1-fold higher signal for the 2dGO fragment (Figure 5C) and a 10.2-fold higher signal for the O6-m2dGO fragment (Figure 5D) compared to methods focused on the parent ion, indicating an early fragmentation occurring during ionization or shortly thereafter. Similarly, the analyses in full scan indicated a 19-fold higher intensity for the 2dGO fragment compared to the parent (Figure 5E), and a 3.6-fold higher intensity for O6-m2dGO fragment compared to the parent (Figure 5F).

Using DI, it appeared that the quantification of 2dGO and O6-m2dGO would be hampered by the intrinsic decay of these compounds in the gas phase. When the decay is very pronounced and the stability of the parent compound is low, the analysis of fragments (purines) may be mandatory in order to reach the highest sensitivity in quantitative assays. Because purines of DNA and RNA bear the same mass, it will be important to consider this information for the sample preparation step, in order to specifically digest DNA instead of both DNA and RNA (e.g., by using DNAse instead of exonucleases for digestion).

### 3.3. Ion Mobility Separation of O6-Methylguanine and N7-Methylguanine

**Figure 6:**
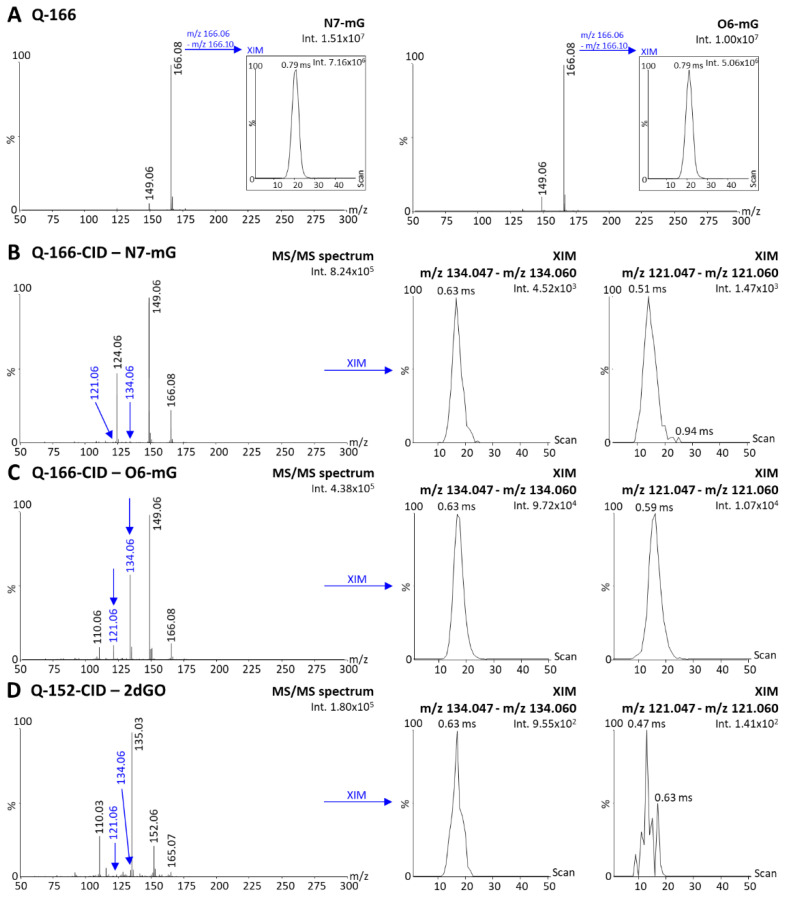
Desorption electrospray ionization (DESI)—ion mobility (IM) mass spectra of N7-methylguanine (N7-mG) and the purine fragment of O6-methyl-2′deoxyguanosine (i.e., O6-mG) acquired with the quadrupole focused on m/z 166 without collision energy (method Q-166) and extracted ion mobilograms (XIM) of the parent peaks (m/z range 166.06–166.10) (**A**). DESI-IM-MS/MS spectra of N7-mG (**B**), O6-mG (**C**), and 2′-deoxyguanosine (2dGO, (**D**)) stock solutions 0.5 µg/mL in MeOH/H_2_O 1:1 acquired with the quadrupole focused on the m/z 166 (N7-mG and O6-mG) or m/z 152 (2dGO) and 20–25 V collision energy for fragmentation (methods Q-166-CID and Q-152-CID). XIM of fragments peaks at m/z 134.06 and m/z 121.06 were extracted from each MS/MS spectrum. Maximum intensities are given in arbitrary units (a.u.) on the top right corner of each spectrum and zoomed view.

An additional parameter to consider while measuring guanine modifications induced by TMZ is the presence of the N7-mG species, which is the most abundant modified species (80 %) with the same mass as the O6-mG species. Any method for the quantification of O6-mG would necessitate to separate these two species to enable specific analyses. Since DI-MS cannot be coupled to liquid chromatography for separation before ionization, an alternative would consist of post-ionization IM separation. In the present context, we investigated whether N7-mG and O6-mG fragments could be separated using the present instrumentation. Since N7-m2dGO is not a commercially available compound, we performed the analyses of N7-mG, which has a parent peak at m/z 166 (Figure 6A, N7-mG), and compared the mobility profiles with the m/z 166 fragment of O6-m2dGO corresponding to O6-mG. The XIM of the m/z 166 peak indicated a similar drift time of 0.79 ms for both N7-mG and O6-mG (Figure 6A); therefore, IM cannot be used for the differentiation of these two species. The last option to differentiate O6 from N7 species would then be the generation of fragments with different m/z and/or DT between guanine, N7-mG, and O6-mG. We, therefore, further fragmented the m/z 166 fragments and two signals appeared as possible signatures of O6-mG that would differentiate it from N7-mG and guanine, m/z 134 and m/z 121 (Figure 6B–D). Both MS/MS and mobility peaks of m/z 134 from O6-mG (Figure 6C) interfered with a peak from both guanine (Figure 6D) and N7-mG (Figure 6B). The peak at m/z 121 and the related DT peak from O6-mG (Figure 6C) did not interfere with any signal from guanine (Figure 6D) but one from N7-mG (Figure 6B). Although the intensity of the peak was 10-fold lower in N7-mG, it is important to note that up to 16-fold higher concentrations of N7-mG can be found in DNA exposed with TMZ. Therefore, minor signal interferences might still represent important hurdles for quantification assays. However, the IM data indicated a slight shift in the maximum DT (ΔDT of 0.08 ms). Although the present IM instrumentation did not provide a clear separation of these two compounds, this is a promising approach for developing quantification assay using instrumentation equipped with ion mobility with higher resolving power.

## 4. Discussion

The development of assays for the quantification of DNA methylations responsible of the biological effect of TMZ (i.e., methylation of guanines at the O6 position) is challenged by the presence of possible sources of interferences from RNA and competitive methylated species induced by TMZ. The development of DI-MS-based methods further adds a layer of difficulty due to the impossibility to separate compounds from the samples before their ionization, e.g., by liquid chromatography. The present characterization of compounds using different DI-MS sources demonstrates that the additional fragmentation, likely due to ISD or early PSD events, further limits the ability to generate quantification assays. However, these observations provide valuable guidance for decisions in initiating developments. First, post-ionization separation of compounds is an instrumental prerequisite to enable the separation of O6-mG from N7-mG species (e.g., using IM instrumentations). In our present context, the IM resolving power of our setup (travelling wave IM spectrometry (TWIMS)) was not sufficient to permit a separation of O6-mG and N7-mG. In addition, the occurrence of fragmentation events should be investigated. In such cases, it should be determined whether it is a quantitatively relevant event and whether it would result in an increase in the lower limit of quantification (LLOQ) of O6-m2dGO above the expected concentrations in the samples. In this case, it should be determined when the fragmentation mainly occurs during the MS analyses, i.e., before or after the quadrupole, in order to set it accordingly. The resolution of quadrupoles is also a critical issue for the development of such quantification assays. Quadrupoles that allow for high-resolution mass selectivity without loss of sensitivity (e.g., in a triple quadrupole) allow for the secure selection of 2dGO from DNA and the exclusion of guanosines (GO) originating from RNA. In a situation where increasing the resolution of quadrupoles reduces analytical sensitivity, the 17-Da molecular weight difference between 2dGO and GO and between O6-m2dGO and O6-mGO may be critical for their analysis with high selectivity. In this case, the sample preparation must be adapted to target digestion only to DNA and not to RNA (e.g., with DNAases).

**Figure 7:**
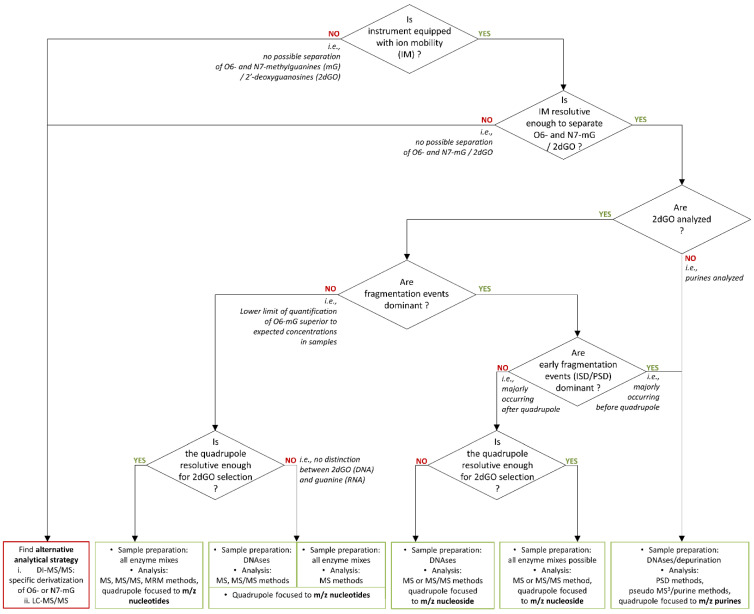
Decision algorithm for method development of a monitoring assay of temozolomide action through the quantification of O6-methylguanines (O6-mG) or O6-methyl-2′-deoxyguanosine (O6-m2dGO).

Based on this knowledge, several options would be available. This is illustrated in Figure 7, which can be used as a guide for future decisions to initiate the development of assays for the monitoring of DNA modifications induced by TMZ. Although in our present context, the IM resolution of TWIMS was not sufficient for the separation of O6-mG and N7-mG, other IM equipment, such as trapped IM spectrometry (TIMS), might provide higher mobility resolving power for a better separation. Preliminary results in our context indicated that IM separation might be more efficient on pseudo-MS3 fragments of O6 and N7-methylated purines than on ISD/PSD fragments of methylated purines. The IM separation of pseudo-MS3 fragments will, however, not be possible with instrumentations having the mobility cell before the quadrupole and collision cells, e.g., with the timsTOFflex (Bruker Daltonics, Billerica, USA) [31]. The success of the separation of O6 and N7-methylated species would rely on the IM separation of methylated guanosine species.

It is noteworthy that Kimura and co-workers observed ISD effects in MALDI on oligonucleotides with nucleobase derivatives, which may be attributed to the structural proximity and close contact between the matrix and the analyte [32]. It could be hypothesized that ISD/PSD events could be due to a concentration effect of the compounds in the deposits with a close proximity of the compounds to one another. This effect might be reduced in biological matrices, when nucleosides are present at low concentrations and mixed with endogenous compounds. Preliminary results of electrospray ionization (ESI)-MS and the development of assays of cell culture models currently indicate that (i) fragmentation events also take place with ESI but can be attenuated with specific source-tuning, such as lower cone voltage, which seems to prove the predominance of ISD events in the analyte fragmentation and that (ii) exposure to TMZ can lead to concentrations of O6-mG in the pg/µg tissue range, which represent low concentrations for DI-MS-based analyses. These assumptions and observations may lead to additional recommendations for workflows before investigating O6-mG concentrations by DI-MS analyses:

(1) Determine by LC-MS/MS the concentration of O6-mG in the established biological model of TMZ exposure.
(2) Analyze the determined concentration of O6-mG in the biological matrix of interest by DI-MS to evaluate whether (i) fragmentation events take place and where they do in the MS and (ii) whether the concentration can be set as the LLOQ.

## 5. Conclusions

The analysis of the pharmacodynamic methylation effects of TMZ is a complex task, especially using DI-MS, which is hampered by several possible artefacts. Here, we provide important information to avoid pitfalls in DI-based analyses of O6-mG induced by DNA exposure to TMZ.

## Figures and Tables

**Table 1:** Description of the applied mass spectrometric methods.

Method Name	Quadrupole (Q) Focusing	Collision-Induced Dissociation (CID)	Related Method in [20]
**Full scan**	No	No	Method 2
**Q-268**	Yes, m/z 268	No	Method 3
**Q-282**	Yes, m/z 282	No	Method 3
**Q-152**	Yes, m/z 152	No	Method 3
**Q-166**	Yes, m/z 166	No	Method 3
**Q-152-CID**	Yes, m/z 152	Yes, 32 V	Method 4
**Q-166-CID**	Yes, m/z 166	Yes, 32 V	Method 4

## Data Availability

The data presented in this study are available on request from the corresponding author.
